# Individual risk perception and empirical social structures shape the dynamics of infectious disease outbreaks

**DOI:** 10.1371/journal.pcbi.1009760

**Published:** 2022-02-16

**Authors:** Valeria d’Andrea, Riccardo Gallotti, Nicola Castaldo, Manlio De Domenico

**Affiliations:** 1 CoMuNe Lab, Fondazione Bruno Kessler, Trento, Italy; 2 Department of Physics and Astronomy “G. Galilei”, University of Padova, Padova, Italy; University of Zaragoza: Universidad de Zaragoza, SPAIN

## Abstract

The dynamics of a spreading disease and individual behavioral changes are entangled processes that have to be addressed together in order to effectively manage an outbreak. Here, we relate individual risk perception to the adoption of a specific set of control measures, as obtained from an extensive large-scale survey performed via Facebook—involving more than 500,000 respondents from 64 countries—showing that there is a “one-to-one” relationship between perceived epidemic risk and compliance with a set of mitigation rules. We then develop a mathematical model for the spreading of a disease—sharing epidemiological features with COVID-19—that explicitly takes into account non-compliant individual behaviors and evaluates the impact of a population fraction of infectious risk-deniers on the epidemic dynamics. Our modeling study grounds on a wide set of structures, including both synthetic and more than 180 real-world contact patterns, to evaluate, in realistic scenarios, how network features typical of human interaction patterns impact the spread of a disease. In both synthetic and real contact patterns we find that epidemic spreading is hindered for decreasing population fractions of risk-denier individuals. From empirical contact patterns we demonstrate that connectivity heterogeneity and group structure significantly affect the peak of hospitalized population: higher modularity and heterogeneity of social contacts are linked to lower peaks at a fixed fraction of risk-denier individuals while, at the same time, such features increase the relative impact on hospitalizations with respect to the case where everyone correctly perceive the risks.

## Author summary

The spreading of a disease across a population is affected by the compliance with behavioral restrictions, enforced by governments to slow the diffusion of an epidemic. In this study, we use a large-scale survey to relate compliance with behavioral rules to individual level of disease risk perception. We asses that absence of risk awareness is associated with a set of harmful behaviors (namely, non-compliance with: social distancing, use of facial masks and adoption of any prevention measures) that can accelerate the diffusion of an epidemic. Through a mathematical model, we study how epidemic dynamics, and in particular hospitalization burden, is affected by the presence of different fractions of the total population who do not correctly perceive the disease risk and, accordingly, adopt harmful behaviors. Moreover, we study how different social contact structures among individuals modulate the effect on epidemic spreading of a fixed population fraction with null risk perception. Our findings highlight that a fixed percentage of people with null risk awareness has a lower impact on epidemic size in social structures characterized by communities and heterogeneity in contacts among individuals. The spreading of a disease across a population is affected by the compliance with behavioral restrictions, enforced by governments to slow the diffusion of an epidemic. In this study, we use a large-scale survey to relate compliance with behavioral rules to individual level of disease risk perception. We asses that absence of risk awareness is associated with a set of harmful behaviors (namely, non-compliance with: social distancing, use of facial masks and adoption of any prevention measures) that can accelerate the diffusion of an epidemic. Through a mathematical model, we study how epidemic dynamics, and in particular hospitalization burden, is affected by the presence of different fractions of the total population who do not correctly perceive the disease risk and, accordingly, adopt harmful behaviors. Moreover, we study how different social contact structures among individuals modulate the effect on epidemic spreading of a fixed population fraction with null risk perception. Our findings highlight that a fixed percentage of people with null risk awareness has a lower effectiveness on epidemic size in social structures characterized by communities and heterogeneity in contacts among individuals. However, in these same social structures, larger fractions of risk-denying population cause an enhanced effect on epidemic size.

## Introduction

Severe acute respiratory syndrome (SARS) in 2003, Middle East respiratory syndrome (MERS) in 2012 and COVID-19 in 2020 are examples of highly infectious diseases for which no pharmaceutical treatments such as drugs or vaccine were readily available at the time of their outbreaks. In such cases, non-pharmaceutical interventions (NPIs) have been the most important resource to contain the epidemics and effective tools in the outbreak management [1–6].

In the case of COVID-19, the global fight against the spread of the SARS-CoV-2 virus is heavily relying on a large number of NPIs which, for most countries, were based on significant behavioral changes at individual and collective level, enforced by governments in order to mitigate the diffusion of the epidemics [7–12]. The corresponding sudden change in habits and practices has naturally faced, across the globe, a wide range of levels of public acceptance.

Even if compliance with specific control measures was crucial to delay the disease spreading, the adoption of behavioral changes is far from being homogeneous across a population and individual choices are affected by epidemic risk perception [13–18].

To better understand how the people’s knowledge, beliefs, behaviors, risk perception and compliance with required rules differ around the world, a research group led by the MIT designed a survey [19] consisting of 20 multiple choice questions (see MIT survey data section in Materials and methods). The survey has been conducted over a large sample of Facebook users across 64 countries and replicated 13 times during a time-span of six months between July 2020 and January 2021. Across all countries, the survey covers in total an effective sample size of over half a million subjects with a minimal effective sample size over a country of 500.

In this work we investigate the impact of people with null risk perception (here named risk-deniers) on epidemic spreading. We first identify a set of behaviors that is robustly related to a low or null level of individual risk perception, like non-compliance with social distancing and with use of facial masks. We then develop a mathematical model with compartmental states that explicitly take into account the adoption of the selected mitigation behaviours. We study effects of different amounts of non-compliant, risk-denier people on the evolution of the epidemics on top of synthetic models characterized by increasing complexity (e.g., level of clustering, modular structure) and on 187 real-world networks. We find evidence that modular structure and degree heterogeneity have a twofold effect on epidemic spreading, as dynamics on top of networks characterized by those topological features show a lower peak of hospital admissions but also an hospitalization amount that is more strongly affected by increasing quantities of risk-deniers people.

## Results

We analyze the aggregated results of the MIT survey, with the goal of identifying sets of beliefs and behaviors which systematically appear together. The aggregated survey results provide information about the fraction of respondents to 140 nonexclusive alternative answers for each of the 64 countries. Given this abundance of information, we decided to analyze the correlations across the whole data set by means of UMAP clustering technique (see, in Materials and methods, section Dimensionality reduction and correlation analysis for further details).

**Fig 1:**
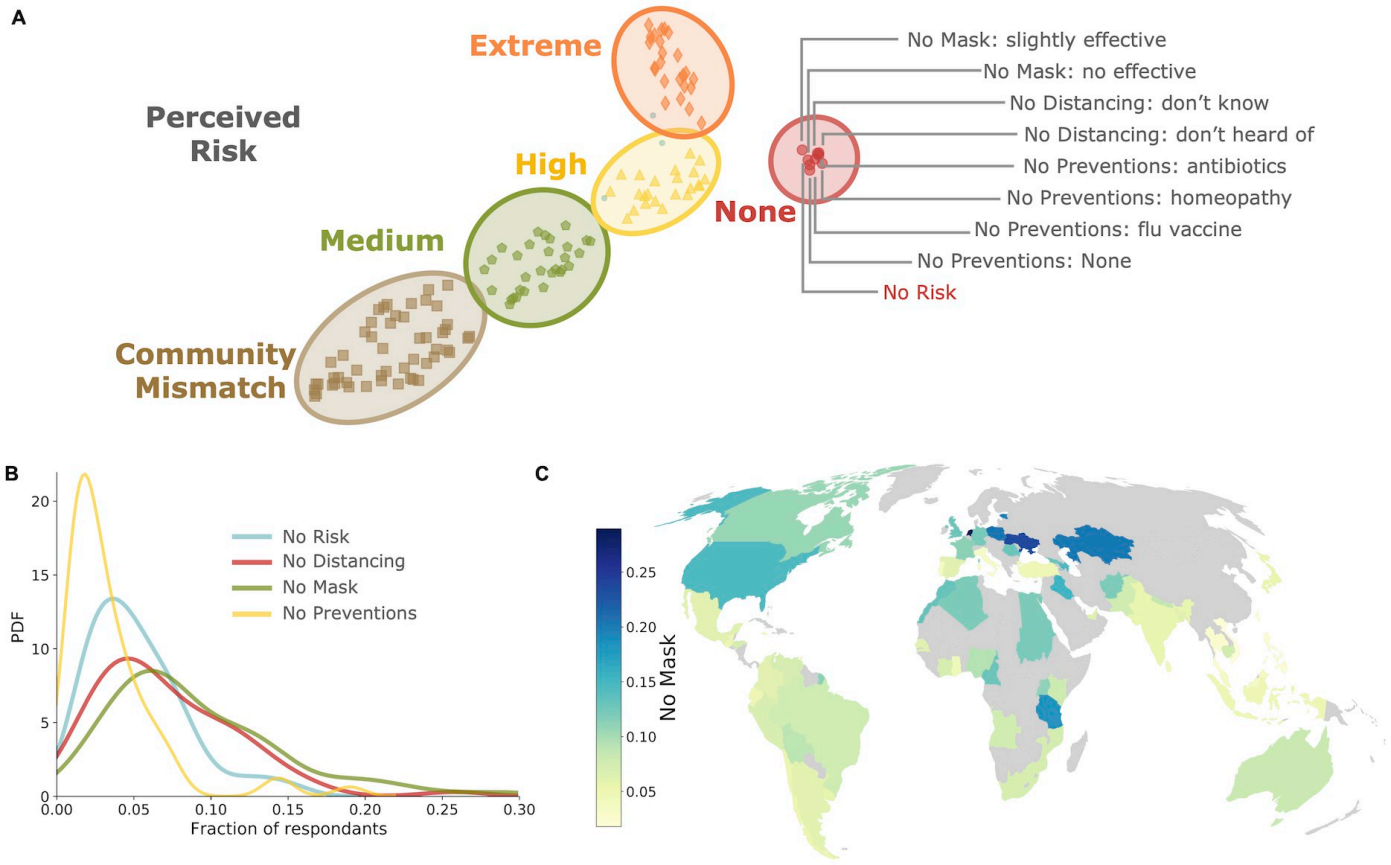
Isolating behaviors associated with null risk perception in the MIT COVID-19 beliefs, behaviors & norms survey. a: UMAP embedding and HDBSCAN clustering. Using UMAP for dimensionality reduction, projecting the multidimensional manifold where the survey data lie to two dimensions and the clustering algorithms HDBSCAN to identify questions in the survey where globally answers were similar, we isolate in the survey a set of non-compliant behaviors (red circles) associated with the lack of perception of danger associated with COVID-19. Other clusters include answers indicating higher level of perceived danger (gray pentagons, yellow triangles and orange diamonds, see Materials and methods) or with a mismatch between the importance for the respondents of taking action against the epidemics and the perceived importance for the community (brown squares). b: Fraction of respondents who declare non-compliant behaviors in the 64 countries considered. Besides the community risk (blue), all answers of the “No risk” cluster of panel (a) are associated with three questions isolating three non-compliant behaviors: unfamiliarity with social distancing (red), disbelief in face mask effectiveness (green) and the lack of preventive actions taken (yellow). Together with the lack of actions taken, also other ineffective actions are included in the “No risk” cluster, but they are here not aggregated together with the “None” answer since the answers were non-exclusives. c: World map of the fraction of respondents doubting the effectiveness of masks. Higher mistrust in mask usage is observed in Africa while Asian countries appear to be more accustomed to the public health control measure. Map dataset from Natural Earth website (https://www.naturalearthdata.com/). See also S2 Fig for maps associated with the other three questions.

In Fig 1A we show that different clusters can be distinguished by the answer to one of the most prominent questions posed, evaluating the level of risk perceived in the community (“*How dangerous do you think the COVID-19 risk is to your community?*”). The most isolated cluster collects 9 answers labelled in the figure as “No risk”, as it includes the response “*Not at all dangerous*” to the aforementioned question, thus identifying a set of answers associated with the belief that there is no risk associate with the COVID-19. These answers are associated to only other three questions:

- *Which of the following best describes your familiarity with the term “physical distancing” during the COVID-19 pandemic?* (ANSWERS: *I have heard of it but do not know what it means / I have not heard of it*);
- *How effective is wearing a face mask for preventing the spread of COVID-19?* (ANSWERS: *Not effective at all / Slightly effective*);
- *What measures have you taken to prevent infection from COVID-19 in the past week?* (ANSWERS: *None / Getting the flu vaccine / Using antibiotics / Using homeopathic remedies*).

These selection of answers therefore isolate three behaviors associated with the belief that COVID-19 is not dangerous. A person convinced that COVID-19 is not dangerous: i) does not comply to social distancing rules, poorly understanding the concept of physical distancing; ii) does not believe that face masks are effective; iii) does not act to prevent infection, or acts as it was just a flu. These behaviors clearly deviate from the rules necessarily enforced by the public health authorities to mitigate the COVID-19 epidemic, representing the non-compliance behaviors we explicitly consider in our epidemic model, in the remainder of this study. (Robustness of clustering results is evaluated in S1 Fig).

Once isolating these non-compliant behaviors, we quantify the population fraction of non-compliant individuals in the countries covered. In Fig 1B, we illustrate the distribution of fractions in all countries for the “No risk” community (which distribution display a peak at about 5%), those of the non-compliance with social distancing (“No distancing”, peak at 6%) and not believing in the effectiveness of face masks (“No mask”, peak at 7%). Given how the survey is designed, it is not possible to quantify the fraction of respondents who answered not to take any meaningful prevention against the infection, but we can identify a lower bound as the fraction of users who answered “None” (“No preventions”) which has a peak at 3% in the survey. The same distribution of the fraction of respondents is mapped in Fig 1C, where is illustrated the fraction of individuals who do not believe in face masks effectiveness, and in S2 Fig where we illustrate the fraction of individuals who do not perceive risks and do not understand social distancing. We find that 34% of the countries has a percentage of non-compliance with the use of masks higher than 10%, and among them several high income countries such as the United States of America, United Kingdom, France, Germany, Canada and the Netherlands. The distributions refer to average values computed over six months. For 23 countries, the survey has been regularly repeated every two weeks over a total of 13 waves of online survey: by using these time series, one can also study the evolution in time of these non-compliant behaviors. In S3 Fig we illustrate the example of the belief that face masks are ineffective in preventing the spread of the virus. The evolution of this belief is highly variable across waves and countries, with values that can exceed 30% and, on average across all the countries, the peak value is 8%. Similarly the peak values of waves, estimated as averages across countries for “No risk”, “No distancing” and “No preventions”, are 10%, 12% and 8%, respectively.

The role played by human behavior in the spread of an infectious disease has been widely investigated by means of mathematical models that usually describe both opinion and epidemic dynamics, as well as the interactions between those two processes ([20–22]). Here we explore the effect of behavior using an *ad hoc* generalization of the compartmental susceptible-exposed-infectious-recovered (SEIR) model, which explicitly accounts for non-compliant infectious people (ID) as one of the epidemic states (see Epidemic model). In our hypothesis, people belonging to the ID compartment represent risk-denier infectious people that, according to results from the survey, do not comply to social distancing, do not use face masks and, in general, do not adopt any measure to prevent the diffusion of the infection. The adoption of such control measures is, in our model, realized only by compliant infectious people (IC). The isolation compartment (Q) is the epidemic state modeling the adoption of mitigation behaviours. We derive the dynamics of population fractions in each compartment by using an agent-based approach that explicitly takes into account the non-trivial connectivity structure among individuals. We test our epidemic model on 4 distinct types of synthetic network models and 187 real networks belonging to 9 different datasets.

Synthetic models with distinct topological features are selected to gain insights into how specific network features, typical of human interaction patterns, may affect the epidemic spreading. Specifically, we run our analysis on Erdős-Rényi (ER) networks, where links are drawn uniformly at random, small world (WS) networks, exhibiting high transitivity and low average path length, Barabási-Albert model (BA), characterized by a highly heterogeneous connectivity distribution, and a stochastic block model (SBM4) characterized by modular structure resembling communities. As reported in S4 Fig, we validate our model assumptions by comparing solutions of mean-field equations with stochastic simulations. As expected, analytical solutions provide a good description of dynamics on BA and ER networks, whereas mismatch with simulations is observed when networks are characterized by modular structures. Therefore, we opted to rely on agent-based simulations for subsequent analysis, although the mean-field approximation will be used later to compare predicted hospitalized amount with observations in the USA at the state level (see S6 Fig).

In order to quantify the epidemiological impact, we characterize the epidemic dynamics through the maximum value (peak) along the time course of hospitalized population (H). This measure is estimated using fixed epidemic parameters in our model, while varying the population fraction of infectious risk-deniers *α* between *α* = 0, corresponding to a scenario where all the infected people are compliant with the mitigation rules, and *α* = 1, simulating a scenario where all infected people are risk-deniers. Evaluating the basic reproduction number *R*_0_ can itself support our hypothesis that non-compliant behaviours have an impact on epidemic size. In particular, we estimate *R*_0_ and we use the closed-form equation that describe peak of infectious people as a function of *R*_0_ to gain insights on the increase with *α*. Theoretical estimates are confirmed by stochastic simulations and provide a lower bound (see S5 Fig for details).

To test the goodness of our model in a real scenario, we compare predictions on hospitalized admissions to observed values in a set of countries. In order to avoid confounding factors we test our model in a time window at which MIT survey data are available and interventions put in place to reduce transmission (like full closure of nonessential businesses, restrictions of mobility or stay-at-home orders) remain unchanged. We simulated epidemic dynamics in the different USA states on a time period between July 6 and September 27, 2020. Epidemic model parameters are the same described in Epidemic model, with the exception of the fraction of risk-deniers, that is inferred, for each country, from MIT survey data. In our model, we also set values at *t* = 0 as the total population, number of infectious, hospitalized and recovered people in each country reported in ‘the COVID tracking project’ data set [23]. We run our model using mean-field approximation, estimate the fraction of hospitalized population and compare those values with real hospitalized population reported in USA states. Results are shown in S6 Fig, where we also report Spearman rank correlation coefficient and p value, respectively *ρ* = 0.78 and *p* = 3 × 10^−10^. Correlation analysis suggests that the proposed model is able to capture epidemic trend, but further information (like, for example, age-structured contact rates or more detailed socioeconomic data) should be considered to reproduce real epidemic data.

**Fig 2:**
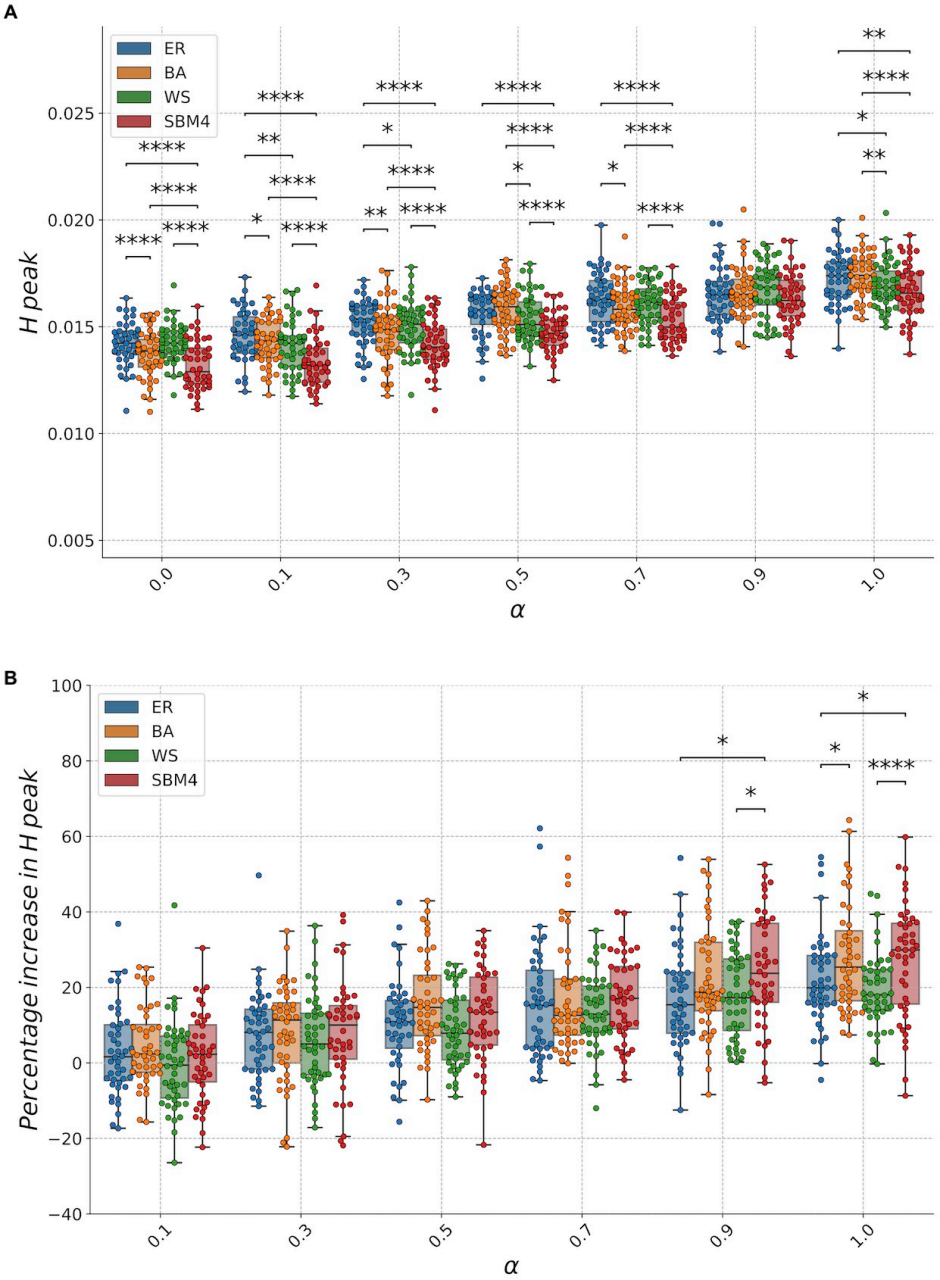
Population fraction of risk-deniers affects epidemics in synthetic networks models. a: Peak of hospitalized population as a function of the fraction of population of infectious risk-deniers *α*, for, respectively, uniformly random (ER), Barabási-Albert (BA), Watts and Strogatz (WS) and stochastic block model (SBM4). Each dot represents the average measure across 50 dynamical samples of a single network realization, box-plots show quartiles of distributions across 50 network realizations. We perform a Kruskal-Wallis H-test to test the null hypothesis that the population medians at fixed *α* are equal. Post hoc pairwise comparisons between groups at fixed *α* are required to determine which distributions are different. To this aim, for those *α* values with Kruskal-Wallis H-test p value *p* ≤ 0.05, we use post-hoc pairwise comparisons between distributions. In particular we use a Mann-Whitney test, because groups are independent, corrected for multiple comparisons. Significance results are reported as: ****: *p* ≤ 10^−4^, ***: 10^−4^ < *p* ≤ 10^−3^, **: 10^−3^ < *p* ≤ 10^−2^, *: 10^−2^ < *p* ≤ 0.05. Results show that the presence of communities, as in SBM4, significantly decreases the hospitalized peak for all *α* fractions, except for *α* = 0.9. b: Peak of hospitalized population evaluated with respect to the one estimated at *α* = 0.0, as percentage increase. At high fractions of risk-deniers (*α* ≥ 0.9), community structures give rise to higher increase in hospitalizations peak with respect models with different topology.

**Fig 3:**
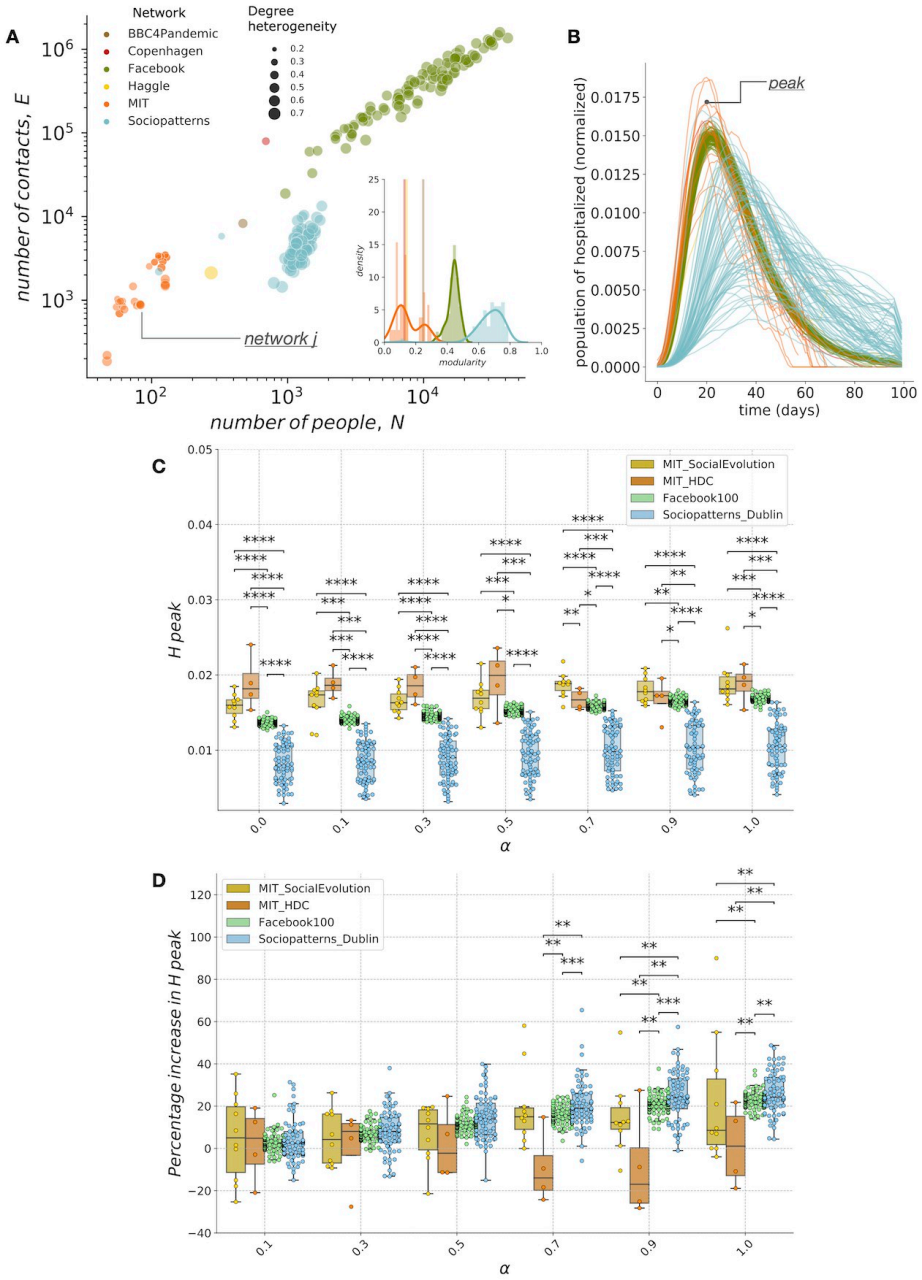
Population fraction of risk-deniers affects epidemics in real networks. a: Scatter plot describing number of contacts (edges, E) as a function of the number of individuals (nodes, N) for real-world social network data set (n = 187 networks) considered in this study. Each dot is a single network, its color identifies the database source and its size is proportional to the connectivity heterogeneity measured by the Gini coefficient (see Synthetic and real-world network data for further details). Inset: distributions of modularity show that the considered data sets of real social networks spans from structures characterized by the presence of communities (high modularity) to structures with loosely connected communities (low modularity). b: Temporal evolution of the population of hospitalized patients in real networks. Model dynamics is evaluated at a fixed fraction (*α* = 0.5) of risk-deniers. Color identifies the database source as shown in panel (a). Values of the population at its maximum height correspond to the peak. c: Peak of hospitalized patients as a function of the population fraction of infectious risk-deniers *α* for data sets with multiple network instances. Each dot represents results in a single network, box-plots show quartiles of distributions in each data set. Statistical tests and significances as in Fig 2. Results show that networks characterized by high values of degree heterogeneity and modularity, like the ones in Sociopatterns data set, show a peak of hospitalized patients that is significantly lower than peak evaluated in other real networks. d: Peak of hospitalized patients evaluated with respect to the one estimated at *α* = 0.0, as percentage increase. At high fractions of risk-deniers (*α* ≥ 0.7), data sets characterized by community structures and high heterogeneity values show an higher increase in hospitalizations peak with respect data set with low modularity and heterogeneity values.

Impact on epidemic spreading caused by different levels of *α* across multiple synthetic networks is summarized in Fig 2A, where we show the peak evaluated from hospitalized population time course. (Examples of temporal evolution of hospitalized people in real world networks are shown in Fig 3B)). We find that maximum hospital burden increases when we increase the population fraction of risk-denier infectious people, for all the considered network models. Furthermore, our results show that, when compared to other network models at fixed *α*, SBM4 has an hospitalized peak that is significantly lower (Kruskal-Wallis H-test followed by post-hoc pairwise comparisons with Mann-Whitney test corrected for multiple comparisons). Our results are in agreement with previous studies that attested how community structure has a hindering effect on spreading for both total size and peak height ([24]). To gain insights into the trend of hospitalized peak as a function of the fraction of risk-deniers, we evaluate the percentage increase in hospitalized peak at different *α* values with respect to the one estimated at *α* = 0.0, as shown in Fig 2B. Our results reveal that at high population fractions of risk-deniers, modular structure of individual contacts, as in SBM4, significantly enhances the effects of non-compliance with rules on hospitalization peak.

We study the evolution of the epidemic on top of more than 180 real-world networks, where nodes are individuals and links are either physical contacts or a proxy for them. Fig 3A shows that the real social network data set spans more than 3 orders of magnitude in number of individuals and more than 4 orders of magnitude in number of contacts among individuals. Moreover both heterogeneity in the number of contacts, here measured using Gini coefficients (see Synthetic and real-world network data) and modularity distribution cover a wide spectrum of possible values. Fig 3B displays time course of hospitalized population in the considered real networks, where we set the fraction of risk-deniers at *α* = 0.5 to simulate dynamics. In Fig 3C are shown, at different *α* values, distributions of hospitalized peak for data sets with multiple network samples, namely the two data sets from MIT, the Facebook friendship data set and Dublin Sociopatterns data set (see Synthetic and real-world network data for further details). Our results show that the higher are the values of modularity and heterogeneity that characterize a specific data set (from the lowest to the greatest: MIT, Facebook and Sociopatterns), the lower is the evaluated hospitalized peak. On the other hand, as shown in Fig 3D, data sets with higher modularity and heterogeneity display, at high *α* values, a greater percentage of peak increase. In other words, presence of community structures and degree heterogeneity can enforce the effect that risk-deniers individuals have on epidemic size.

**Fig 4:**
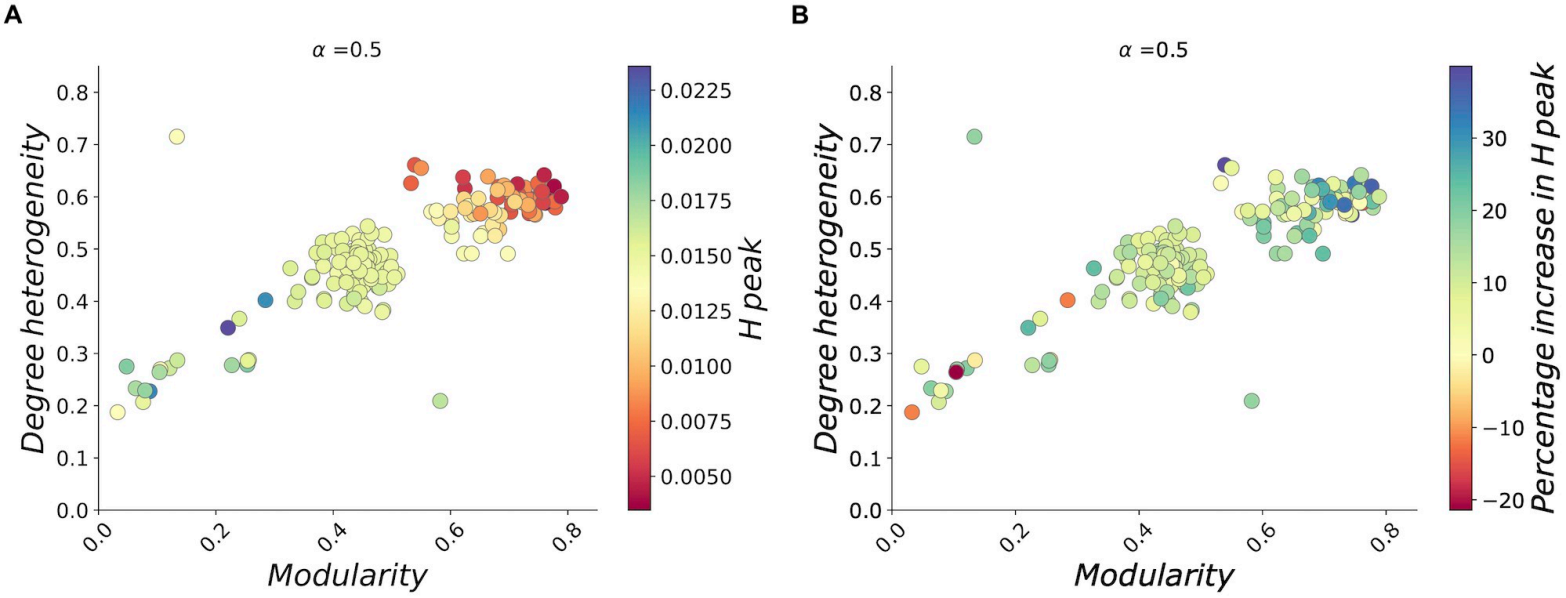
Degree heterogeneity and community structure affect hospitalized peak in real networks. Each dot represents degree heterogeneity as a function of modularity for each real network considered in this study. Model dynamics is evaluated at fixed population fraction of risk-deniers (*α* = 0.5) a: Peak of hospitalized patients. Results show that the lower are values of modularity and heterogeneity, the higher is hospitalized peak. b: Peak of hospitalized patients evaluated with respect to the one estimated at *α* = 0.0, as percentage increase. Networks with high modularity and heterogeneity show higher percentage peak increase.

The latter hypothesis is further supported by the results in Fig 4, where are shown results for all real-world contact patterns considered in this study, without any classification according to the data set to which they belong. The position of each point is a function of network modularity and degree heterogeneity, while color encodes hospitalized peak and increase in hospitalized peak with respect *α* = 0 for, respectively, panel a) and b). Here we obtain epidemic dynamics by setting the fraction of risk-denier population to *α* = 0.5. Scatterplots show that, the higher are network heterogeneity and modularity, the lower is the peak of hospitalized patients and the higher is the percentage increase of the hospitalized peak with respect the scenario with zero risk-deniers. In other words, considered together, heterogeneity in degree distribution and community structure affect in opposite ways absolute epidemic size and risk-deniers effect on epidemic size. In Fig 4A we show that, at fixed percentage of risk-denier population, hospitalized peak is hindered in epidemic processes taken place on networks with high modularity and heterogeneity in connectivity patterns. As reported previously in this paper, clustering can slow the spreading of the disease [25], whereas we hypothesize that the hindering effect promoted by degree heterogeneity may be explained by the decrease in network synchronizability postulated in the so called “paradox of heterogeneity” [26, 27]. On the other hand, as shown in Fig 4B, when evaluating effects on hospitalized peak caused by a fraction of risk-deniers with respect to the scenario with no deniers in the population, we find that increment in hospitalized peak is significantly greater for epidemic processes on networks with modular structures and high levels of heterogeneity in the number of contacts.

## Discussion

Our findings address the problem of how much disease risk perception can quantitatively affect epidemic spreading on networks with topological features typical of human interaction patterns. To this aim we design a compartmental model where we explicitly account for behaviors that we found are related to null risk perception. In our model study we assume that infectious people, whatever is their risk perception and compliance with rules, spread the disease. Therefore, in our model, we are considering a group of infectious people that is bigger than the real world one with the result of underestimating the effects of risk-deniers infectious people variations.

A similar underestimation happens also in the case of the pool of susceptible people because compliant people will be more likely to adopt preventive measures (e.g. wearing masks, avoiding social gatherings, etc).

Therefore, in our model we are effectively considering the worst-case scenario where the pool of susceptible individuals is maximum (due to the fact that we don’t consider NPIs adoption in the susceptible compartment) and where the pool of infectious individuals maximizes the probability to spread the disease. A possible improvement to the model would be to split the susceptible compartment in two different sub-populations (ideally, an *SC* and an *SD* compartment) according to people compliance to mitigation rules, as we did for the infectious population.

In this work, epidemic dynamics is estimated using fixed parameters reported in a recent epidemiological model of COVID-19. It is worth noting that all the results here shown depend on the chosen parameters and that our model should be tested with other sets of parameters, extracted from other references, in order to investigate the robustness of our results.

According to survey results, fractions of respondents who declare non-compliant behaviours are highly variable across different countries and different epidemic waves, with values that can exceed 30%. Nevertheless, considering mean values over time, non-compliance with specific mitigation rules, such as the use of facial masks, is higher than 10% in several countries. These values coming from social-media based surveys are naturally prone to a selection bias [28] towards highly educated people. The level of education is expected to be weekly associated to risk-denial, as highly educated people might be slightly more prone to adopt protective behavior [29]. This would eventually skew the values recorded towards a lower representation of risk-deniers in the online survey. Our analysis evaluated on real-world networks highlights that the observed level of risk denial drive an increase of about 3.5% for median and about 7% for the third quartile, for the total amount of hospitalized patients.

Therefore, the level of non-compliance with mitigation rules deduced from MIT survey has, as a percentage increase, a low impact for national health systems. Nevertheless, it is worth noting that the economic cost associated with relatively low percentage increases in hospitalized individuals can be remarkable.

To give an example for USA, where non-compliance with use of facial mask is higher than 10%, it was estimated that there were about 850, 000 hospitalizations due to COVID-19 in a period of almost a year (51 weeks, from March 8, 2020 to February 27, 2021 [30]). It was also estimated that the average cost of treatment for an inpatient admission for a patient without insurance is 73, 000*US*$ [31], though it is important to note that high costs could be due to the extreme price inflation characteristic of the US insurance system. If we assume the percentage increase of hospitalized patients of 3.5%, it would be equivalent to an increase of more than 2 billion US dollars. A similar analysis for United Kingdom, where there were about 400, 000 hospitalizations and where the average cost of a COVID-19 hospital stay is about 6, 700*US*$ [32], would lead to a cost increase of more than 100 million American dollars due to non-compliant behaviours. The lack of publicly available information and systematic data about this type of costs for each country makes difficult a more thorough analysis, but it is plausible to suppose that the economic burden for European and other OECD countries is of the same order of magnitude.

For COVID-19, despite the encouraging vaccination campaigns, non-pharmaceutical interventions still provide the most effective mitigation strategies, thus requiring the highest percentage of compliance at both individual and community levels, although the same applies for other emerging diseases. Information on ongoing disease outbreaks travels fast, often in an uncontrolled, incomplete or conflicting way which makes the ground for the infodemic phenomenon [33, 34], as pointed out by the World Health Organization [35], a cause of concerns during the COVID-19 pandemic [36–40]. Our findings demonstrate that different social contact structures produce different (and non-linear) responses to the same amount of risk-denier individuals. Consequently, in the next future, it will be critical to account for both human connectivity patterns and risk perception when designing mitigation strategies based on non-pharmaceutical interventions.

## Materials and methods

### Synthetic and real-world network data

We run simulations of our epidemic model on both synthetic and real-world networks. Synthetic networks are here used to both validate our model and to better understand results on real networks. Artificial networks are generated with some of the most widely used models of network structure, namely: scale free (Barabási-Albert, BA), uniformly random Erdős-Rényi (ER), stochastic block model (SBM4) and small world (Watts and Strogatz, WS) model. For each model we generated 50 network realizations with *n* = 256 nodes and average degree 〈*k*〉 = 12 and we run our epidemic models over 50 dynamics samples.

We also study the epidemic dynamics on 187 real-world networks, collecting from 9 different data sets, that report contact structures between individuals. Contacts between individuals are both virtual, reporting Facebook friendship network (Facebook100 [41] and part of Copenhagen [42] data sets), and real, when identifying physical proximity among participants (Copenhagen, BBC4Pandemic [43, 44], Haggle [45], MIT [46, 47], Sociopatterns [48–50]). It is worth noting that, in this work, virtual contacts between individuals are used as a proxy of physical contact patterns. That is certainly a limitation of our framework, for example because a friendship connection between two individuals on a online network does not necessarily mean that there is also a connection between the two individuals in the physical network, that is the one relevant to the spread of infectious diseases. Furthermore online social networks could also miss some contact relevant for the transmission of the disease.

Nevertheless, to address the specific purposes of this work, to choose virtual contacts as a proxy of physical interactions could be appropriate because we focused our analysis on the topological features that affects epidemic spreading. Previous works found that online communities have very similar structural characteristics to offline physical contact networks [51, 52]. Furthermore, some of the data sets used in this work, such as the Copenhagen and the Sociopatterns ones, have been used to model epidemic spreading in several works [53–55].

It is also worth noting that actual physical interaction were identified via Bluetooth or GPS signals in all real-world data sets with the exception of the Sociopatterns data set where, in 74/75 of networks, proximity is estimated using Radio-Frequency-Identification-Devices (RFID) embedded in conference badges of conference participants and museum visitors.

We evaluate heterogeneity of real-world network structures using the Gini coefficient: $G=1-\sum_{i=1}^{N}[1/N(2\sum_{k=1}^{i}W_{k}-W_{i})]$, where *W*_*i*_ is the ratio of *i*th node’s degree to the total degree of all nodes and 0 ≤ *G* ≤ 1 [56].

### MIT survey data

The MIT survey [19] has been performed through Facebook across 64 countries in biweekely waves starting from July 2020. In this work we use answers collected between July 2020 and January 2021. The total number of respondents per country ranges between 500 and 30.000. We aggregate over all age, genders and education level to obtain a single value per country. The survey includes 20 questions, including questions with a single mutually exclusive answer such as:

- If a vaccine for COVID-19 becomes available, would you choose to get vaccinated?
- How dangerous do you think the COVID-19 risk is to your community?
- Which of the following best describes your familiarity with the term ‘physical distancing’ during the COVID-19 pandemic?
- How effective is wearing a face mask for preventing the spread of COVID-19?
- How often are you able to wear a mask or face covering when you are in public?

…

On the other hand, there are also a number of questions where multiple answers are possible such as:

- In the past week, from which of the following, if any, have you received news and information about COVID-19?
- Which of the following businesses, locations, or events would you visit or attend in the coming two weeks if they were operating at full capacity?
- What measures have you taken to prevent infection from COVID-19 in the past week?

…

The question “How dangerous do you think the COVID-19 risk is to your community?” is the one that we use for labeling Fig 1, together with the “community mismatch” indicating the difference between the answers to “How important is it for you to take actions to prevent the spread of COVID-19 in your community?” and “How important do other people in your community think it is to take actions to prevent the spread of COVID-19?”. The labeling has been made for sake of illustration: as can be seen in S1 Fig clusters vary between different UMAP seeds, with the exception of the one that identifies non-compliant behaviors.

### Epidemic model

In this work we use a compartmental model that classify an individual according to both his epidemic state and his behaviour. Our model included the following compartments: susceptible (S) individuals who have not yet contracted the disease, exposed (E) individuals who are not yet contagious, infectious with compliant behaviors (IC) who mitigate the spreading of the disease by adopting ad-hoc interventions, infectious with non-compliant behaviors, or risk-denier infectious (ID) who don’t adopt measures to contrast disease spreading, hospitalized (H) people who require hospitalization, self-isolating or quarantined (Q) infectious individuals who stay at home and are compliant with NPIs (wash hands, use masks, etc) to stop the spreading of the disease, recovered (R) people who are no more infectious. All the infectious people, regardless of their behaviour, leave their compartments and may reach H or R compartments, but only infectious people IC, compliant with distancing and use of masks, can reach the Q compartment. The equations describing the dynamics the of disease transmission within each population are given by:

$$\begin{array}{c} \begin{array}{cc} & \dot{S}=-\lambda S\\ & \dot{E}=\lambda S-\sigma E\\ & \dot{IC}=\sigma \left(1-\alpha \right)E-\delta aIC-\eta bIC-\gamma \left(1-a-b\right)IC\\ & \dot{ID}=\sigma \alpha E-\delta aID-\gamma \left(1-a\right)ID\\ & \dot{H}=\delta a\left[IC+ID\right]-\gamma_{hosp}H\\ & \dot{Q}=\eta bIC-\gamma Q\\ & \dot{R}=\gamma \left(1-a-b\right)IC+\gamma \left(1-a\right)ID+\gamma_{hosp}H+\gamma Q \end{array} \end{array}$$ (1)

where:

$$\begin{array}{c} S+E+IC+ID+H+Q+R=1 \end{array}$$ (2)

**Fig 5:**
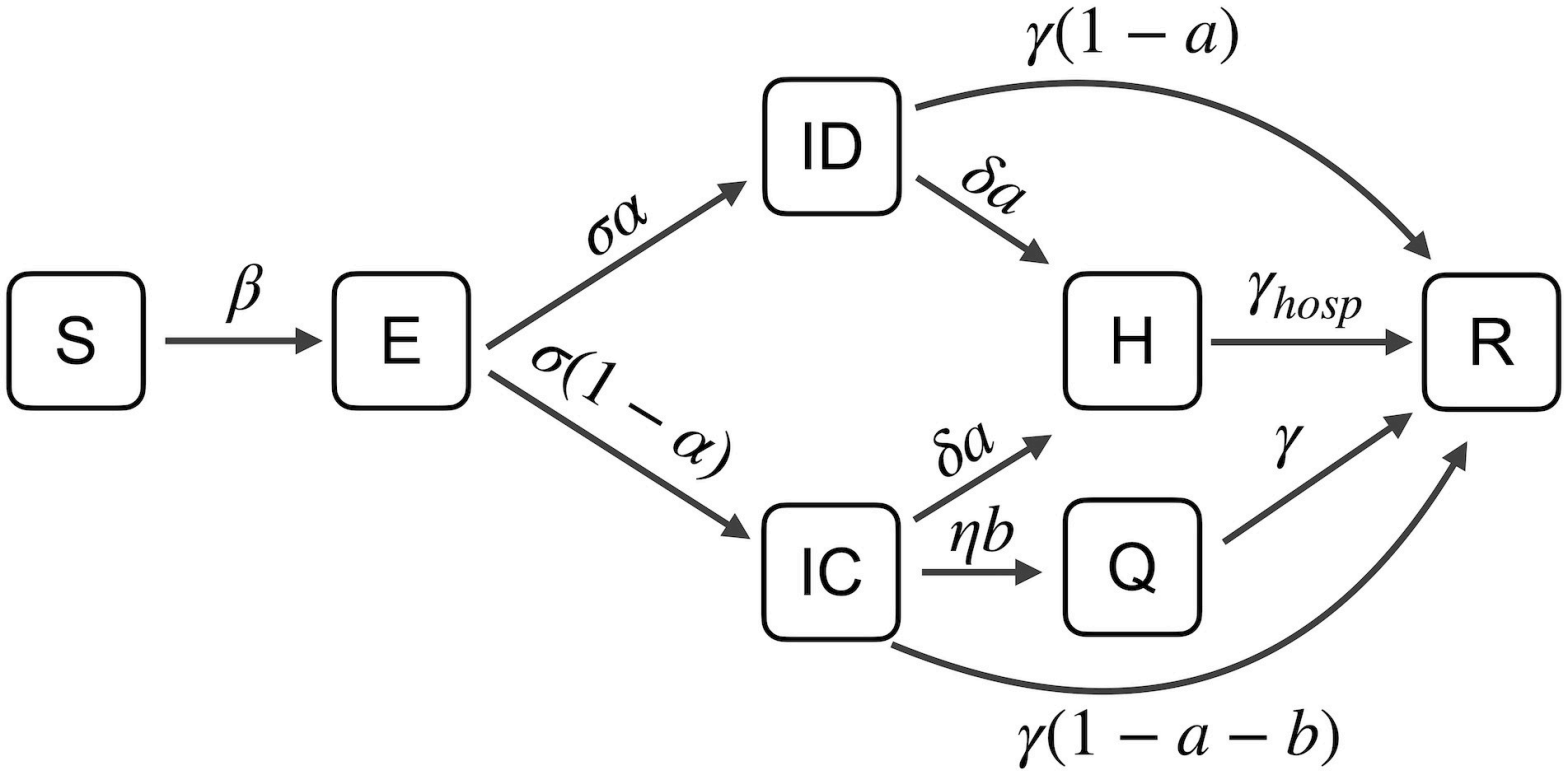
Graphical scheme of the epidemic model. Compartments included in the model are: susceptible S, uninfected individuals, exposed E, individuals who are not yet contagious, infectious with compliant behaviors IC, who mitigate the spreading of the disease by adopting NPIs, infectious with non-compliant behaviors, or risk-denier infectious ID, individuals who are not compliant with mitigation rules, hospitalized H, people who require hospitalization, self-isolating or quarantined Q, infectious individuals who stay at home and are compliant with NPIs, recovered R, people who are no more infectious. Model parameters are: force of infection λ, incubation rate *σ*, recovery rate *γ*, recovery rate for hospitalized patients *γ*_*hosp*_, hospitalization rate *δ*, quarantine rate *η*, fraction of risk-deniers *α*, fraction of population that needs hospitalization *a* and fraction of population that are quarantined at home *b*.

Each variable is the fraction of individuals in a specific compartment at time *t* and parameters are force of infection (λ), incubation rate (*σ*), recovery rate (*γ*), recovery rate for hospitalized patients (*γ*_*hosp*_), hospitalization rate (*δ*), quarantine rate (*η*), fraction of risk-deniers (*α*), fraction of population that needs hospitalization (*a*) and fraction of population that are tested as positive for the disease and are quarantined at home (*b*). A scheme of the epidemic model is shown in Fig 5. To derive the dynamics, in this work we set the values of the parameters to the ones recently estimated for epidemiological models of COVID-19 [57]. In particular, we set the expected amount of people one infectious individual can infect per day as: infection transmission rate *β* = 0.5, *σ* = 1/6 *day*^−1^, *γ* = 1/7 *day*^−1^, *γ*_*hosp*_ = 1/14 *day*^−1^, *δ* = 0.025 *day*^−1^, *η* = 0.33 *day*^−1^, *a* = 0.2, *b* = 0.2.

### Simulation of epidemic dynamics and validation

We use two approaches to analyze the dynamics of the population in each compartment, and then we compare results to validate our methods. We simulate the dynamics by taking into account the stochastic nature and the network connectivity structure through which epidemics is transmitted among individuals by using a Gillespie algorithm (GA) [58], that decompose the dynamics into independent spontaneous processes and then perform a change of state of each node by a non fixed time step. We impose an initial condition of a single infectious individual, that we assigned to IC or ID compartment with a probability depending on *α* and for each synthetic network realization and for each real network we simulate 50 dynamic samples. We also adopt a deterministic analytical framework by solving the ODE systems in the homogeneous mixing hypothesis, that do not require the knowledge of the precise contact network but assumes that all individuals have identical probability of infecting contacts. We are able to relate the dynamics on networks and in the homogeneous mixing approach by writing the probability that a individual on a network is infected in a time interval Δ*t* as:

$$\begin{array}{c} \lambda \Delta t=1-{\left(1-\beta \Delta t\right)}^{N_{k}}=1-{\left(1-\beta \Delta t\right)}^{\frac{\langle k\rangle I}{2}} \end{array}$$ (3)

where (1 − *β*Δ*t*) is the probability that the node is not infected in the time interval Δ*t*, *N*_*k*_ is the number of edges emanating from all infected vertices *I* = *IC* + *ID* and 〈*k*〉 is the average network degree. If *β*Δ*t* ≪ 1, we can approximate $\lambda \approx \frac{\beta \langle k\rangle I}{2}=\tilde{\beta }I$ and obtain a force of infection that does not depend on time interval. In our study we integrate model equations by using both expressions of λ.

### Closed form equations for the basic reproduction number *R*_0_

The basic reproduction number *R*_0_ is the number of secondary infections produced by a single infection in a completely susceptible population and is a good estimate of how fast a disease can spread in a population ([59, 60]). We estimate *R*_0_ using the next generation operator method ([61]) and we define the next generation matrix **G** as the square matrix in which the *ij*th element is the expected number of secondary infections in population *i* caused by an infectious individuals of population *j*. Matrix **G** is in turn composed by the matrix *F* of new infections and matrix *V* of the remaining transfers of infections from one compartment to another:

$$\begin{array}{c} F=[\begin{array}{ccc} 0 & \tilde{\beta } & \tilde{\beta }\\ 0 & 0 & 0\\ 0 & 0 & 0 \end{array}] \end{array}$$ (4)

$$\begin{array}{c} V=[\begin{array}{ccc} \sigma & 0 & 0\\ -\sigma (1-\alpha ) & A & 0\\ -\sigma \alpha & 0 & B \end{array}] \end{array}$$ (5)

where *A* = *δa* + *ηb* + *γ*(1 − *a* − *b*) and *B* = *δa* + *γ*(1 − *a*). We estimate *R*_0_ as the spectral radius (dominant eigenvalue) of **G** = *FV*^−1^ and we obtain:

$$\begin{array}{c} R_{0}=\frac{\tilde{\beta }}{A}[1+\alpha \frac{b(\eta -\gamma )}{B}] \end{array}$$ (6)

Assuming that our model can be cast to a standard SIR model with a $R_{0}=\tilde{\beta }/\gamma_{eff}$, where *γ*_*eff*_ = *AB*/[*B* + *αb*(*η* − *γ*)] and where the infectious compartment *I* is composed by E, IC and ID compartments. In this framework, we can use the analytical solution of the peak height derived for SIR model ([62, 63]) and we write the equation that relates the maximum population fraction of infectious to basic reproduction number *R*_0_:

$$\begin{array}{c} I_{max}=1+K+K\;\text{ln}\;K, \end{array}$$ (7)

where $K=\frac{1}{R_{0}}$.

### Dimensionality reduction and correlation analysis

To identify correlations among different behaviors and opinions in MIT survey data, we use a method that consist of two passages. First, we reduce the dimensionality of the problem using a dimension reduction technique called Uniform Manifold Approximation and Projection (UMAP) [64]. In a few words, UMAP works by a) approximating a manifold on which the data is assumed to lie; b) constructing a fuzzy simplicial set representation of the approximated manifold; c) searching for a low dimensional projection of the data that has the closest possible equivalent fuzzy topological structure. With respect other dimensionality reduction techniques, such as t-SNE (T-distributed stochastic neighbour embedding), UMAP has a higher processing speed and a faster visualization. As a second step, after we reduced the dimensionality of survey data, we cluster the answers of the survey using HDBSCAN [65], in order to identify answers that were systematically provided together across multiple countries. Since UMAP presents some levels of randomness which are then captured by HDBSCAN, in S1 Fig we illustrate the results provided by other 9 different random seeds. In all cases, the same cluster of 9 con-compliant answers is isolated by HDBSCAN.

## Supporting information

S1 Fig UMAP embedding and HDBSCAN clustering. Using 9 random seeds different by that generating Fig 1A, we systematically observe the presence of the same cluster of nine answers used in the manuscript to isolate non-compliant behavior.
(TIFF)

S2 Fig World maps. a: Fraction of respondents declaring they do think the COVID-19 is dangerous in their community. b: Fraction of respondents declaring they are not familiar with “physical distancing” c: Fraction of respondents declaring they have not taken any action to prevent infection from COVID-19 in the past week. Map dataset from Natural Earth website (https://www.naturalearthdata.com/).
(TIFF)

S3 Fig Temporal evolution of the belief that that face masks are ineffective, for 23 countries. For each country, blue line reports fraction of respondents sharing this belief along 13 waves and orange dotted line is the average across waves. We observe a great variability across both countries and time.
(TIFF)

S4 FigComparison between mean-field solutions and simulations on networks.Population fraction of hospitalized people: average (continuous line) and s.e.m. (shaded area) across 50 samples obtained with stochastic simulations based on Gillespie algorithm on networks. Dashed lines are the solutions of ODE system in mixed population approximation: black dashed line refer to results obtained with a constant force of infection $\lambda =\tilde{\beta }I$, whereas orange dashed line is solution for a time dependent λ. Panels a, b, c, d refer, respectively, to simulations on BA, ER, SBM4 and WS networks.(TIFF)

S5 FigMaximum population fraction of infectious people at different values: Comparison between agent-based simulations and analytical solution estimated using basic reproduction number.Reported values are percent increases with respect to measures at *α* = 0. Box-plots show quartiles of distributions across 10 ER network realizations, red line is the equation relating infectious peak height to $R_{0}=\tilde{\beta }/\gamma_{eff}$.(TIFF)

S6 Fig Comparison of model predictions with real data. For each USA state, we plot the hospitalized population estimated from our model against the real hospitalized population. Both values describe the situation in a time period between 7/6/2020 and 9/27/2020, as the model is informed by the conditions prior that period and by survey data taken along that timespan. Spearman rank-order correlation coefficient (*ρ*) and p-value are reported.
(TIFF)

S7 Fig Hospitalized peak values evaluated from dynamics modeled at fixed population fraction of risk-deniers. a-b: *α* = 0.1, c-d: *α* = 0.9. Left panels show peak of hospitalized patients, right panels show peak of hospitalized patients evaluated with respect to the one estimated at *α* = 0.0, as percentage increase.
(TIFF)
