# Supplementary figures and images for: Individual risk perception and empirical social structures shape the dynamics of infectious disease outbreaks
Source: PLoS Comput Biol. 2022 Feb 16;18(2):e1009760. doi: 10.1371/journal.pcbi.1009760 (PMC8849607; doi:10.1371/journal.pcbi.1009760)

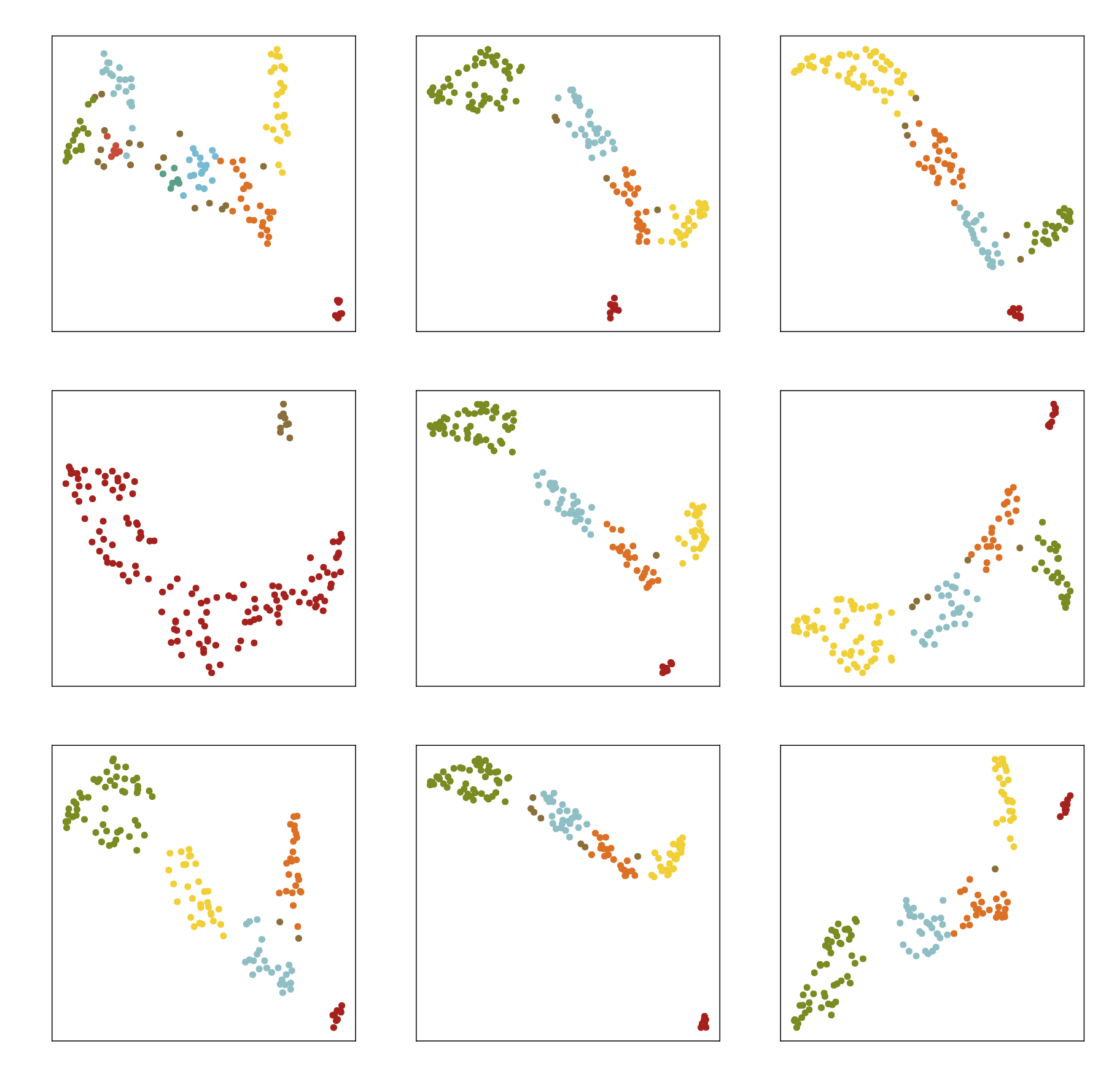

Supplement: S1 Fig — Using 9 random seeds different by that generating Fig 1A, we systematically observe the presence of the same cluster of nine answers used in the manuscript to isolate non-compliant behavior. (TIFF) [file pcbi.1009760.s001.tiff]

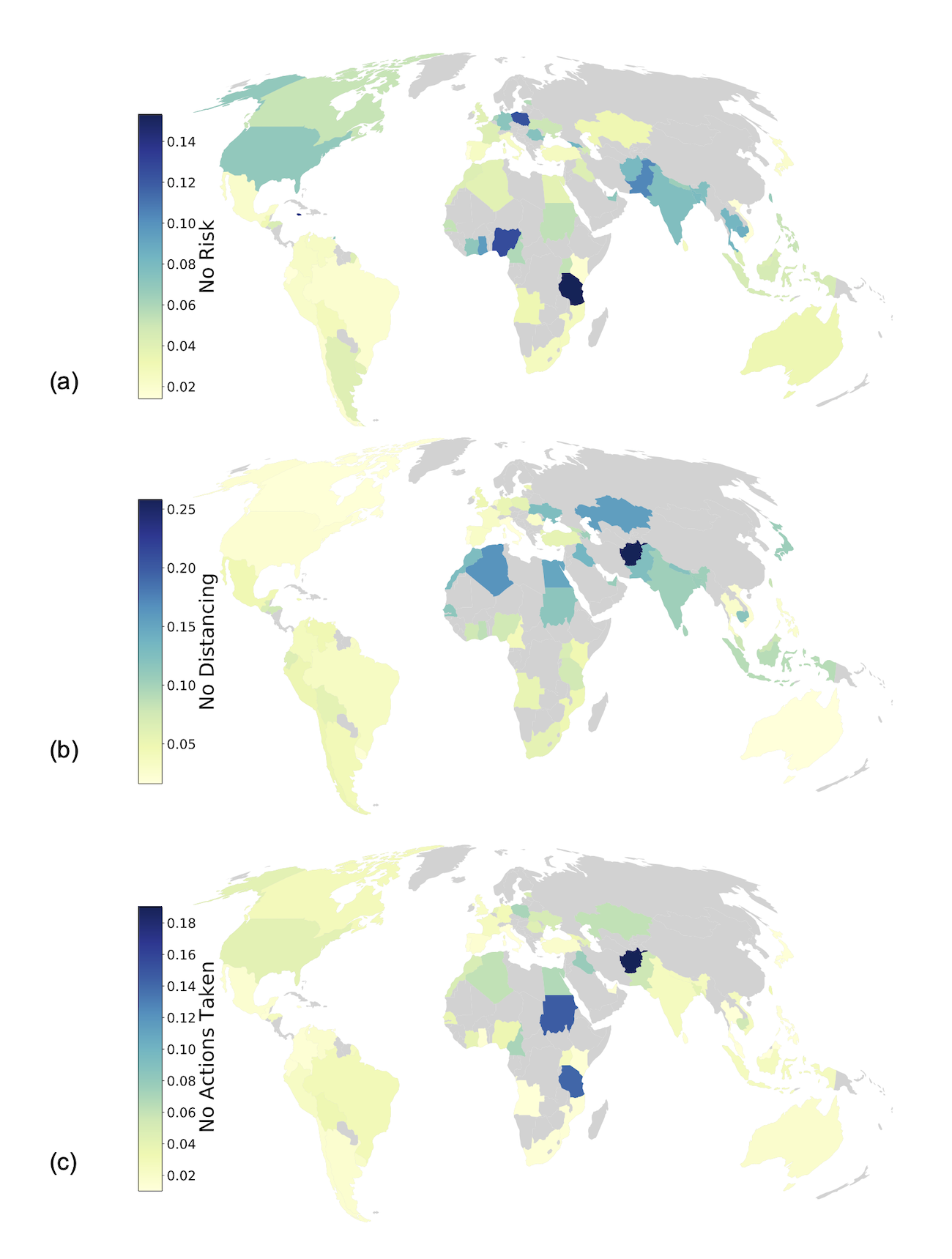

Supplement: S2 Fig — a: Fraction of respondents declaring they do think the COVID-19 is dangerous in their community. b: Fraction of respondents declaring they are not familiar with “physical distancing” c: Fraction of respondents declaring they have not taken any action to prevent infection from COVID-19 in the past week. Map dataset from Natural Earth website (https://www.naturalearthdata.com/). (TIFF) [file pcbi.1009760.s002.tiff]

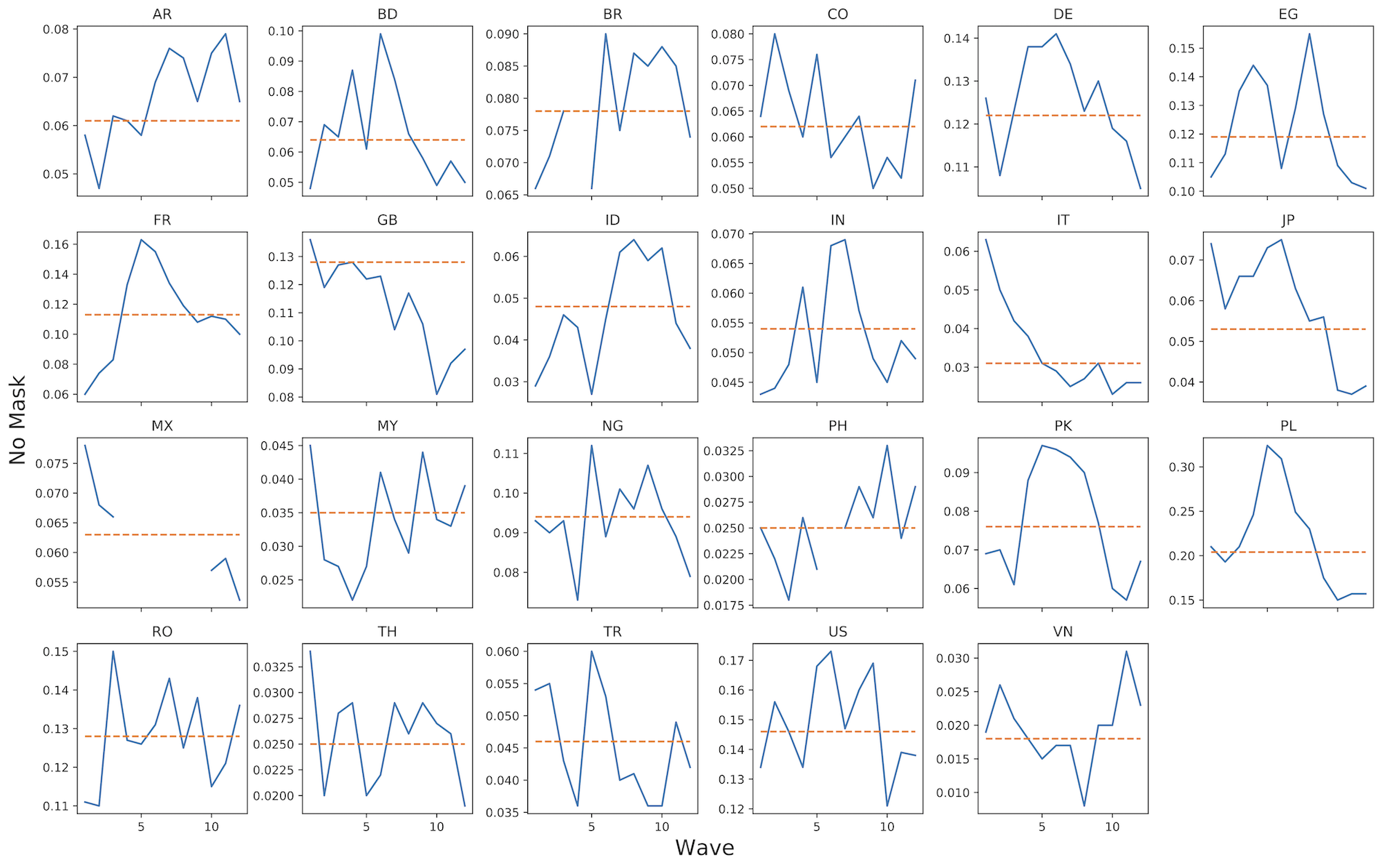

Supplement: S3 Fig — For each country, blue line reports fraction of respondents sharing this belief along 13 waves and orange dotted line is the average across waves. We observe a great variability across both countries and time. (TIFF) [file pcbi.1009760.s003.tiff]

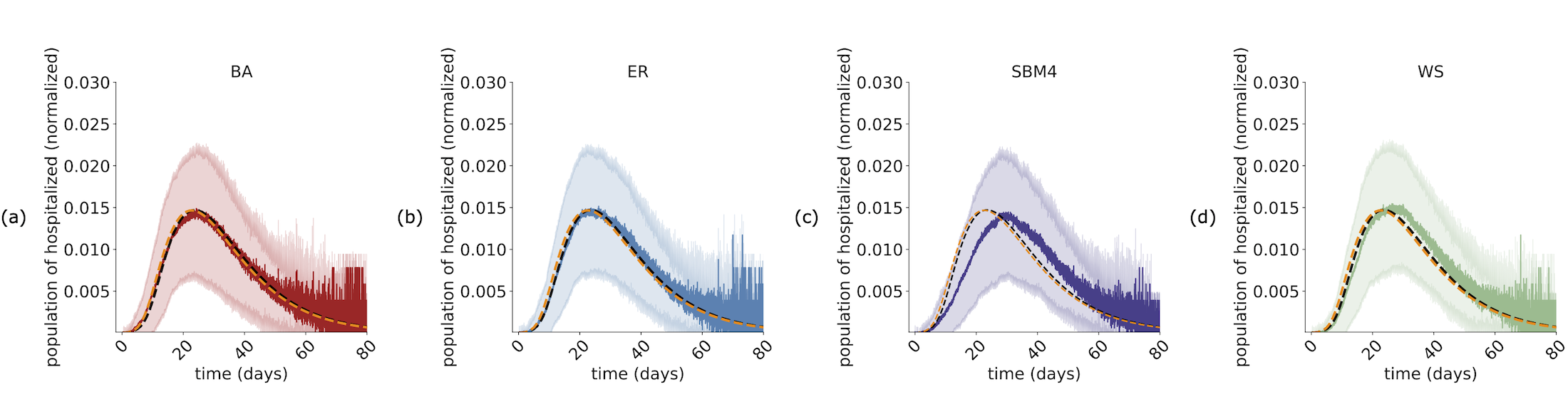

Supplement: S4 Fig — Population fraction of hospitalized people: average (continuous line) and s.e.m. (shaded area) across 50 samples obtained with stochastic simulations based on Gillespie algorithm on networks. Dashed lines are the solutions of ODE system in mixed population approximation: black dashed line refer to results obtained with a constant force of infection λ=β˜I, whereas orange dashed line is solution for a time dependent λ. Panels a, b, c, d refer, respectively, to simulations on BA, ER, SBM4 and WS networks. (TIFF) [file pcbi.1009760.s004.tiff]

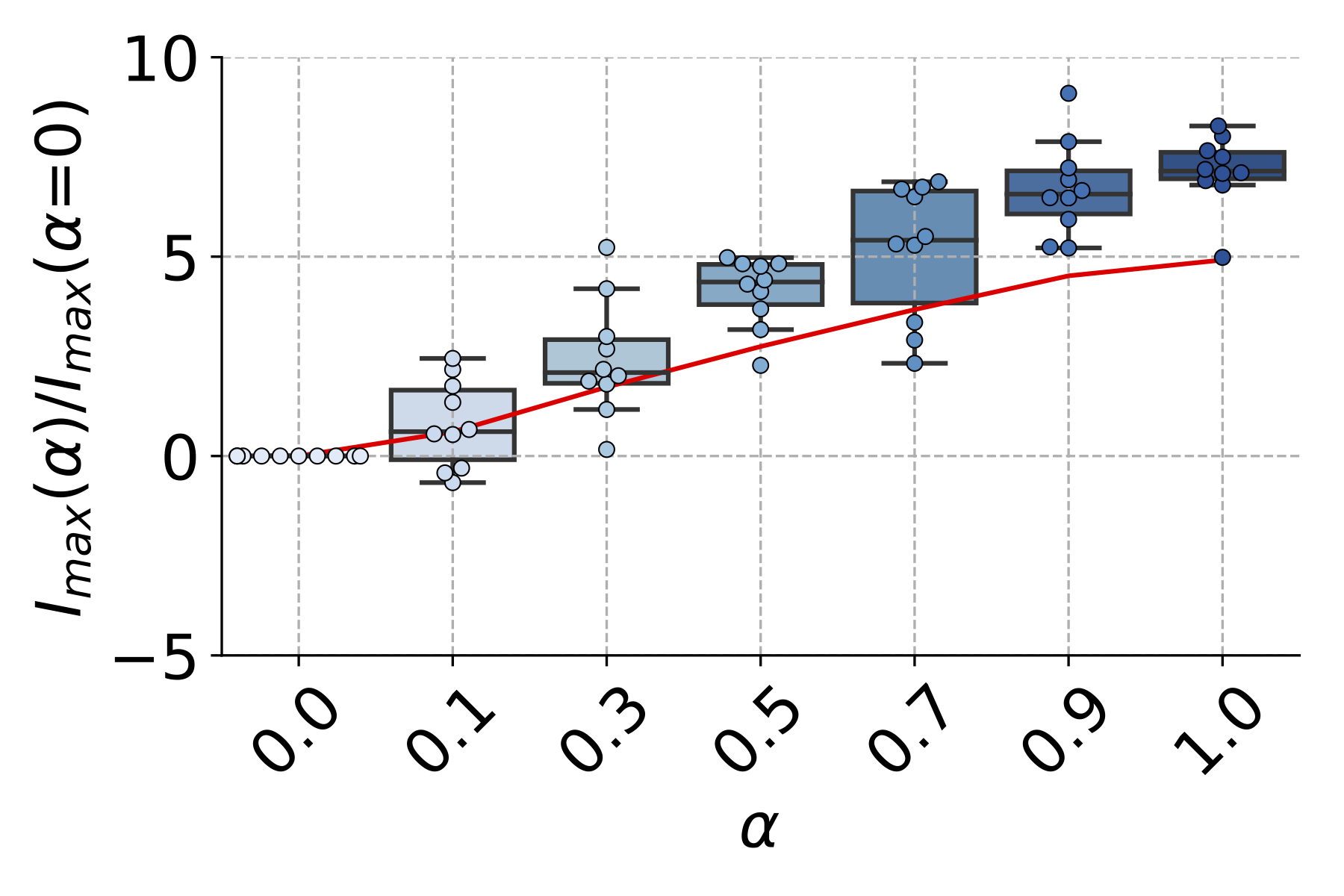

Supplement: S5 Fig — Reported values are percent increases with respect to measures at α = 0. Box-plots show quartiles of distributions across 10 ER network realizations, red line is the equation relating infectious peak height to R0=β˜/γeff. (TIFF) [file pcbi.1009760.s005.tiff]

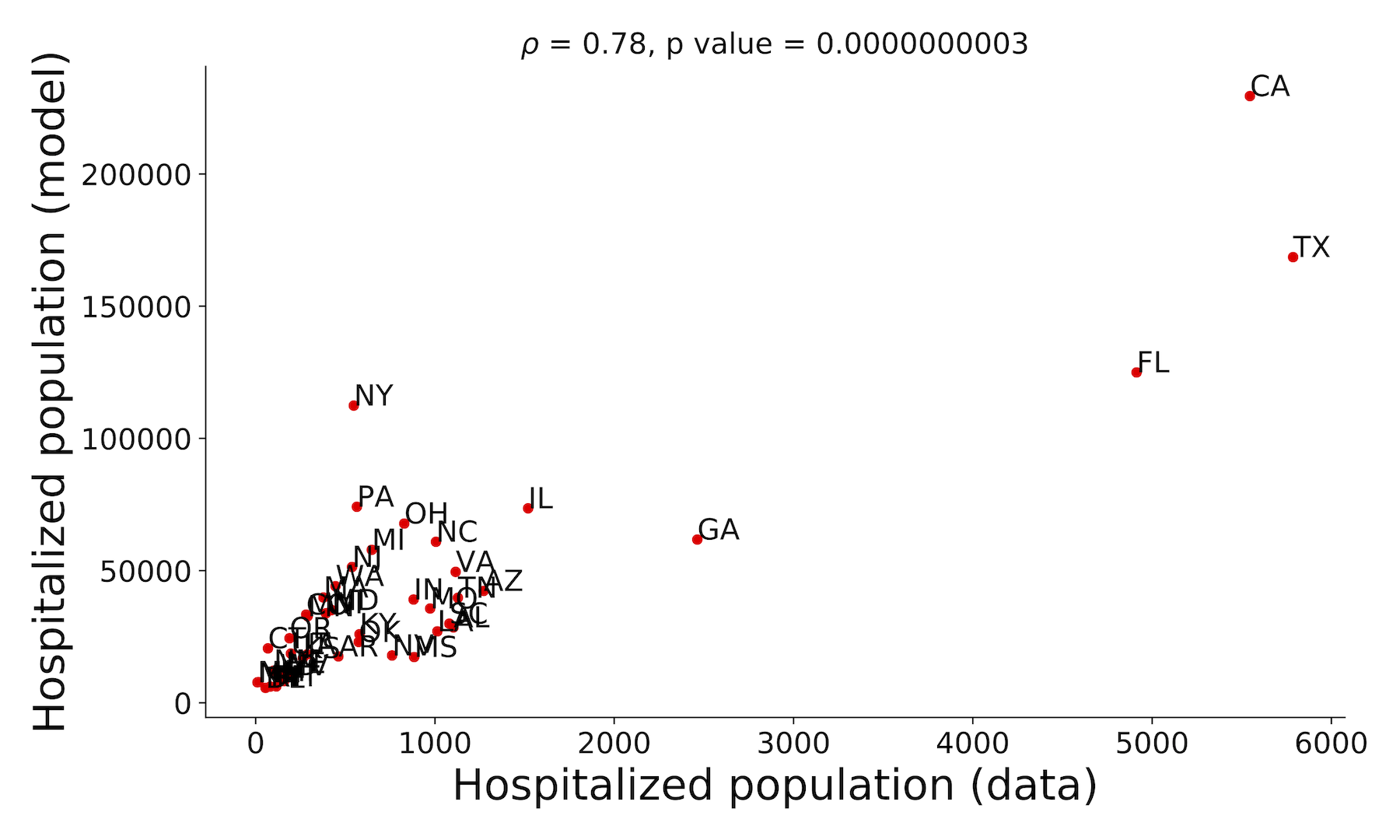

Supplement: S6 Fig — For each USA state, we plot the hospitalized population estimated from our model against the real hospitalized population. Both values describe the situation in a time period between 7/6/2020 and 9/27/2020, as the model is informed by the conditions prior that period and by survey data taken along that timespan. Spearman rank-order correlation coefficient (ρ) and p-value are reported. (TIFF) [file pcbi.1009760.s006.tiff]

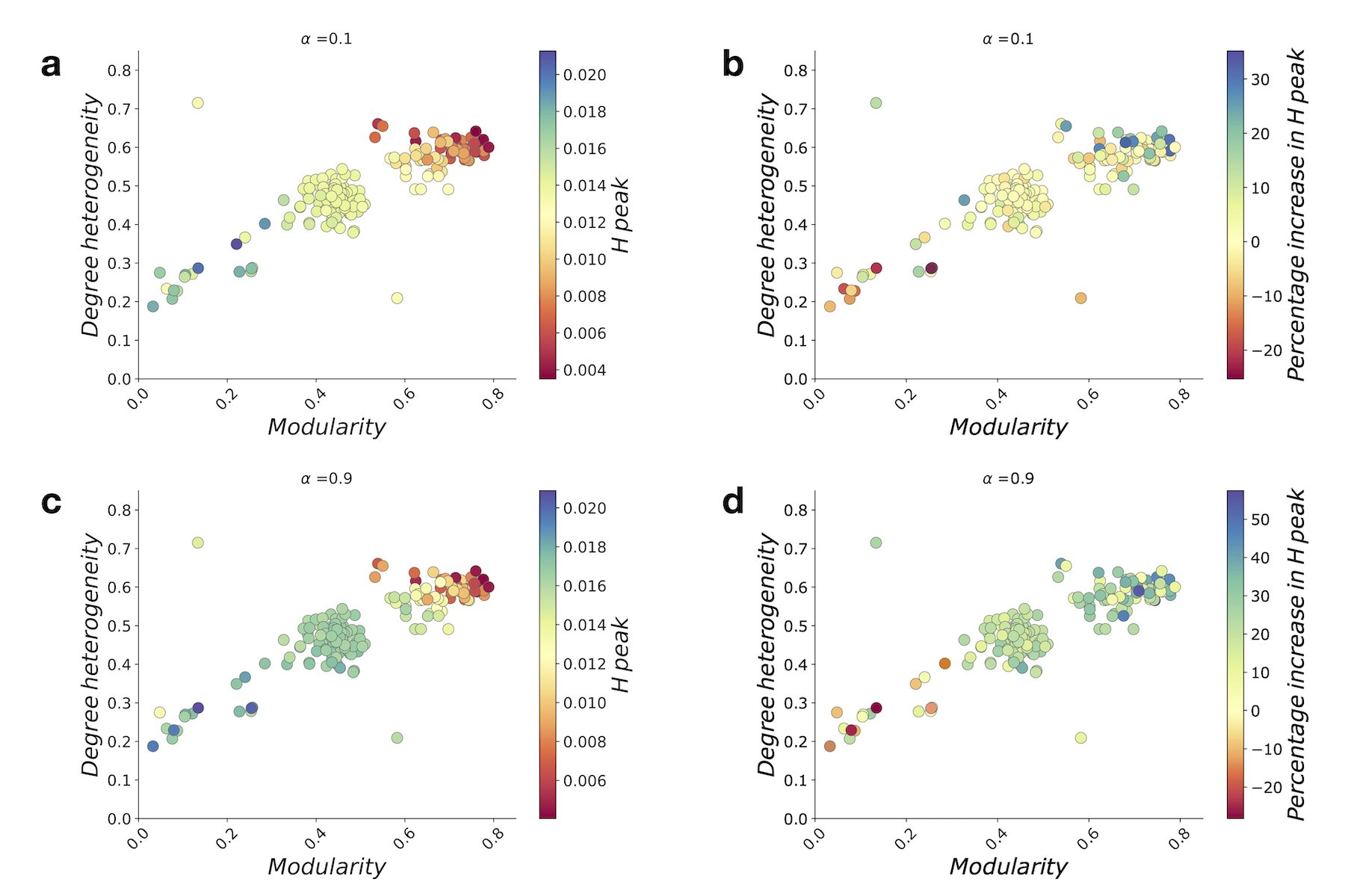

Supplement: S7 Fig — a-b: α = 0.1, c-d: α = 0.9. Left panels show peak of hospitalized patients, right panels show peak of hospitalized patients evaluated with respect to the one estimated at α = 0.0, as percentage increase. (TIFF) [file pcbi.1009760.s007.tiff]
